# Effects of Social Capital on Depression in University Students

**DOI:** 10.3390/ejihpe15050083

**Published:** 2025-05-15

**Authors:** Mario Eduardo Castro Torres, Pablo Marcelo Vargas-Piérola, Aarón Marco Layme Mamani, Andrea Katerine Murillo Toro, Aneydith Ribera Domínguez, Carlos F. Pinto

**Affiliations:** 1 Psychology Department, Universidad San Francisco Xavier, Sucre Casilla 212, Bolivia; mxstercross@gmail.com (A.M.L.M.); murillotoroandrea@gmail.com (A.K.M.T.); aneydith.7@gmail.com (A.R.D.); 2 Independent Researcher, Calle Ladislao Cabrera 482, Sucre 00591, Bolivia; pablomarcelovp@gmail.com; 3 Investigation, Science and Technology Department, Universidad San Francisco Xavier, Sucre Casilla 212, Bolivia; pinto.carlos@usfx.bo

**Keywords:** social capital, socio-emotional support, instrumental support, self-efficacy, self-esteem, academic stress, depression

## Abstract

This study examines how bonding social capital (BSC) is related to depression symptoms (Dsym) in university students, focusing on the mediating roles of socio-emotional support, instrumental support, self-efficacy, self-esteem, and academic stress. A cross-sectional design was employed, with data collected from 217 undergraduate students (from an initial sample of 250) using validated questionnaires. Data were analyzed using partial least squares structural equation modeling (PLS-SEM) to evaluate direct and indirect relationships. The key findings indicate that BSC has an indirect, relevant, and significant negative effect on Dsym (H5) (β = −0.201, 95% CI [−0.266, −0.216]) through six routes, involving enhanced self-esteem and reduced academic stress. The model highlights self-esteem as a critical mediator between social capital and mental health outcomes. With strong predictive validity (R^2^ ≥ 0.1, Q^2^ > 0, PLS-SEM RMSE < LM), the study provides a framework for potential interventions. The theoretical contributions include distinguishing social capital from support and prioritizing self-esteem over self-efficacy in depressive pathways. Although the cross-sectional design limits causal inferences, the model advances systemic approaches to student mental health, highlighting the need for longitudinal validation in diverse contexts.

## 1. Introduction

Studies carried out in recent years have shown a high prevalence of depressive symptoms in university students worldwide, with an average rate of 25% (Akhtar et al., 2020; Arévalo García et al., 2019; Fernandes et al., 2023; W. Li et al., 2022; Paula et al., 2020; Sheldon et al., 2021), which is significantly higher than the general population occurrence (Fernandes et al., 2023).

This elevated prevalence is a highly relevant public health problem, since the continued presence of depression symptoms can lead to more serious mental health troubles (Auerbach et al., 2018; J. Zhang et al., 2024), affecting academic performance (Deng et al., 2022; Y. Wang et al., 2023; J. Zhang et al., 2024), which interferes with the socio-emotional states of students and ultimately diminishes their quality of life (Fernandes et al., 2023), affecting a key population for the social and economic development of any country (Auerbach et al., 2018).

Factors associated with depression in university students include sociodemographic factors, such as type of family; adverse childhood experiences and socioeconomic level; lifestyle factors, i.e., sleep quality, physical activity, substance abuse; and psychosocial factors, i.e., social support, self-esteem, self-efficacy, type of personality, general stress, academic stress and coping styles (Backhaus et al., 2020; Córdova Olivera et al., 2023; Deng et al., 2022; X.-Q. Liu et al., 2022; Mofatteh, 2021; Roldán-Espínola et al., 2024; Yang et al., 2023).

Although the relationship between social capital and health has been studied for more than two decades (Kawachi et al., 2008), only in recent years has the relationship between depression and social capital in university students been investigated, finding negative and significant relationships between these two variables (Backhaus et al., 2020; Backhaus et al., 2022; Sotaquirá et al., 2022; Zhao et al., 2024) and also identifying the same negative relationship between social capital and suicidal ideation—specifically, between several dimensions of social capital (integrated resources, network connection, and trust) and suicidal ideation in university students. However, social participation showed a positive relationship with suicidal ideation (Peng et al., 2019).

The effect of social capital on depression can be explained through three mechanisms: (1) enabling the dissemination and adoption of healthy behavioral standards, as well as the social control of unhealthy behaviors, at a public level; (2) facilitating community accessibility to health-related services and amenities (Kawachi & Berkman, 2014); and (3) allowing access to affective support and acting as a source of self-esteem and the perception of self-efficacy at an individual level (Kawachi & Berkman, 2014; Rosenberg et al., 1995; Thoits, 2011).

Although several studies have shown that social capital has a positive influence on mental health, more specifically over depression, works on this relationship in university students are scarce. Also, there are contradictory results, since some components of social capital may have a negative effect on mental health (Gannon & Roberts, 2020; Mishi et al., 2023; Peng et al., 2019). Additionally, different measurements and definitions of social capital and confusion with other concepts, like social support, do not allow for comparison and proper consolidation of the information provided by previous studies (Adler & Kwon, 2002; Fuertes Eugenio et al., 2013; Mishi et al., 2023). Finally, research on the relationship between social capital and depression has only focused on a direct effect, without considering a model with other variables that have shown a relevant effect on depression, such as socio-emotional support, instrumental support, self-efficacy, self-esteem, and academic stress.

The present study aims to fill these gaps, explaining the relationship between social capital and depression in university students through a model that integrates the most relevant variables involved in this relationship, based on a clear definition of the variables and the use of partial least squares structural equations modeling (PLS-SEM).

### 1.1. Definitions

The variables in the proposed model are defined as follows.

#### 1.1.1. Depression

For the present study, it will be considered as “a negative affective state, ranging from unhappiness and discontent to an extreme feeling of sadness, pessimism, and despondency, that interferes with daily life. Various physical, cognitive, and social changes also tend to co-occur, including altered eating or sleeping habits, lack of energy or motivation, difficulty concentrating or making decisions, and withdrawal from social activities” (VandenBos, 2015, p. 298); that is the main symptom of depressive disorders (American Psychiatric Association, 2013).

#### 1.1.2. Academic Stress

Academic stress is a systemic and adaptive process in which university students evaluate internal demands—expectations based on personal beliefs, goals, and values—and/or external demands—significant people’s expectations, as well as social obligations or limitations—as threats. That is, students consider that they do not have enough resources, neither internal, i.e., emotional self-regulation, knowledge, problem-solving ability, self-efficacy, and self-esteem, nor external—material and/or socio-emotional—to confront the above-mentioned demands (Barraza-Macías, 2018; Lazarus, 2001; Tafet, 2022; Wade-Bohleber et al., 2020).

By perceiving demands as threats, an imbalance that manifests through symptoms, such as psychological (e.g., concentration problems and irritability), behavioral (such as a tendency to argue or dispute), and physical (chronic fatigue), arises in students. (Barraza-Macías, 2018; McEwen & McEwen, 2015; Suárez-Montes & Díaz-Subieta, 2015). Then, students use coping strategies—understood as an affective, behavioral, and cognitive process—focused on activating the resources needed to modify the threat and/or control its consequences (Barraza-Macías, 2018; Lazarus & Folkman, 1984; Lazarus, 2001).

#### 1.1.3. Social Capital

“Social capital can be defined as the sum of tangible—e.g., money, properties, titles—and intangible—e.g., education, political power, social status—resources gathered by having access to a network of reliable, stable and reciprocal social connections. Social capital includes: (1) Available resources embedded in the social network, derived from the exchange process that takes place in it. (2) The social network itself, which is a resource per se. (Bourdieu, 2001; […] Chen et al., 2018; Lin, 2001)”.

(Castro Torres et al., 2023, p. 2)

Depending on the characteristics of the social networks where it is generated, social capital can be divided into two dimensions: (1) bonding social capital and (2) bridging social capital (X. Chen et al., 2009; Putnam, 2001). Likewise, social capital and its dimensions can be measured through (a) the size of the social network, (b) the level of trust towards network members, (c) the degree of access to network resources, and (d) the level of reciprocity perceived in the network (Bourdieu, 1983/2001; Lin, 2001; P. Wang et al., 2014).

#### 1.1.4. Social Support

Social support is understood as “the process (e.g., perception or reception) by which resources in the social structure are brought to bear to meet the functional needs (e.g., instrumental and expressive) in routine and crisis situations” (Lin & Ensel, 1989, p. 383). Social support can be classified as socio-emotional or instrumental, depending on the functional need it seeks to satisfy. The former responds to expressive needs, such as receiving affirmation, displays of affection, appreciation, and/or acceptance, as well as active listening, advice, guidance, and feedback, focusing on emotional well-being and interpersonal validation. In addition, the latter focuses on meeting practical needs by providing material resources (e.g., economic, objects) provision or concrete services (e.g., help with homework, transportation), prioritizing the resolution of tangible problems in specific situations (Buelga & Musitu, 2009; Herrero Olaizola, 2004; Thoits, 1985, 2011).

#### 1.1.5. Difference Between Social Capital and Social Support

The difference between the two concepts lies in their nature and function within interpersonal networks. Social capital refers to accumulated resources (tangible as well as intangible) and the social network structure, understood as an asset that provides access to opportunities, influence, or status. This concept encompasses dimensions, such as network size, trust between its members, and reciprocity, and is classified as “bonding” or “bridging” social capital. In contrast, social support refers to the dynamic process of mobilizing such resources to meet specific needs, whether expressive (e.g., affection and validation) or instrumental (e.g., material aid and services). While social capital represents the “potential” available in the network, social support is the concrete “action” of providing or receiving help, focusing on how those resources are used to deal with everyday or critical situations.

Thereby, the key theoretical contribution of Lin and Ensel (1989) lies in defining social support as a functional process, differentiating its role in stress and health management, which allowed for operationalizing their empirical study. In this line, the studies of Castro Torres et al. (2022, 2023) have demonstrated the relevance of distinguishing between both concepts, evidencing how social capital influences academic stress through mechanisms such as access to resources, while social support acts as a sequential mediator that mobilizes such resources. Their findings underscore the need to validate this distinction, as they confirm that social capital and social support, while interrelated, operate at different analytical levels: one structural and one procedural.

Hence, conceptual clarity between the two concepts is essential for designing effective interventions: social capital can be strengthened by expanding networks and trust, while social support requires strategies for the timely activation of resources, according to individual or collective needs.

#### 1.1.6. Self-Efficacy and Self-Esteem

Perceived self-efficacy is the set of beliefs that each person has about the abilities to plan and execute action plans to manage events that affect own life, according to his/her goals (Bandura, 1993, 1995/2010; Maddux & Kleiman, 2016). “Efficacy beliefs influence how people feel, think, motivate, and behave. Beliefs of self-efficacy produce these various effects through four main processes […]. They include cognitive, motivational, affective and selection processes” (Bandura, 1993, p. 118).

Self-esteem is defined as the positive or negative attitude a person has towards oneself. In such a sense, self-esteem includes a cognitive component (beliefs about oneself) and an affective one (feelings—positive or negative—that such beliefs produce) (Maddux & Kleiman, 2016; Rosenberg et al., 1995).

### 1.2. Research Model

Combining the main effects model and the stress-buffering model of Cohen et al. (2000), this study proposes an integrative multitheoretical model based on Thoits’ (1985, 2011) social support theory. This framework articulates the mechanisms through which bonding social capital (BSC) indirectly mitigates depressive symptoms (Dsym) via sequential psychosocial processes. It is posited that BSC, through socio-emotional support (SES) and instrumental support (IS), provides external social resources that facilitate the development (or strengthening) of internal psychological resources—specifically self-efficacy (sEffic), in line with Bandura’s (1993) social cognitive theory, and self-esteem (sEstee), following Rosenberg et al.’s (1995) theory—consistent with Hobfoll’s (1989) conservation of resources theory (Holmgreen et al., 2017). sEstee influences Dsym either directly or indirectly and is mediated by its impact on academic stress symptoms, as proposed by Barraza-Macías’ (2018) systemic cognitive model, which is, in turn, grounded in Lazarus and Folkman’s (1984) transactional model of stress.

Below, we explain the components of the model, finding each path in previous empirical and theoretical works.

Social capital enables the satisfaction of instrumental needs (requirements of actions and/or resources) and expressive needs (emotional, informational, and expressive requirements) (Bourdieu, 1983/2001; Lin, 2001; Thoits, 1985, 2011). This, in turn, enables the development and/or strengthening of sEffic and sEstee (Kawachi & Berkman, 2014; Rosenberg et al., 1995; Thoits, 1985, 2011).

Social capital enables the satisfaction of instrumental needs (requirement of actions and/or resources) and expressive needs (emotional, information, and expressive requirements) (Bourdieu, 1983/2001; Lin, 2001; Thoits, 1985, 2011), through the process called social support (Lin & Ensel, 1989). This, in turn, enables the development and/or strengthening of self-efficacy and self-esteem (Kawachi & Berkman, 2014; Rosenberg et al., 1995; Thoits, 1985, 2011).

Previous studies have demonstrated that social support is more related to bonding social capital than with bridging social capital (Castro Torres et al., 2022, 2023; Yoo, 2016, 2018). Also, several works have demonstrated a relation between bonding social capital and self-efficacy (Brouwer et al., 2016; Castro Torres et al., 2022, 2023; Y. Liu & Ngai, 2020), between bonding social capital and self-esteem (Aziz et al., 2024; Clark, 2016), and a relationship between social support and self-efficacy (Bayani, 2016; Dela Cruz & Girlie, 2022) and between the latter and self-esteem (Bayani, 2016; Caprara et al., 2013; Fu et al., 2019). Therefore, the following hypotheses are proposed:

**H1.**  *BSC has a direct and positive effect on sEstee*;

**H2.**  *BSC has a direct and positive effect on sEffic*;

**H3.**  *BSC has a direct and positive effect on SES*;

**H4.**  *BSC has an indirect and positive effect on sEstee through the mediation of SES, IS, and sEffic*.

Social capital, specifically, bonding social capital (BSC), influences depression symptoms (Dsym) through its indirect effects on academic stress, specifically, its symptoms, and on sEstee. That is, BSC, through socio-emotional support (SES) and instrumental support (IS), provides social (i.e., external) resources that allow for developing or strengthening psychological resources (i.e., internal, specifically, self-efficacy (sEffic) and sEstee). And sEstee influences Dsym, either directly or through its influence on academic stress symptoms (ASsym) (Thoits, 1985, 2011).

Previous studies have shown that social capital has a direct and negative relationship with depression symptoms (Dsym) in college students (Backhaus et al., 2020; Backhaus et al., 2022; Sotaquirá et al., 2022; Zhao et al., 2024). In addition, previous research has shown that sEstee has a negative relationship with stress symptoms (Backhaus et al., 2020; Bayani, 2016; B. Chen et al., 2024; Deng et al., 2022; Saleh et al., 2017), as well as with depression symptoms (Dsym), in university students (Huang et al., 2023; X. Liu et al., 2024). On the other hand, previous studies have found that social support, self-efficacy, and self-esteem are mediators of the previous variables. For example, the study of Z.-H. Li et al. (2023) demonstrated that sEstee mediates the relationship between psychological capital–which includes hope, resilience, optimism, and self-efficacy–and anxiety in university students. Also, Bayani (2016) showed that sEffic influences test anxiety through the mediation of self-esteem in college students. In addition, Watkins and Hill (2018) found that SES influences Dsym in university students through the mediation of perceived stress. Finally, previous studies have shown that social capital influences mental health in the general population through social support (Mishi et al., 2023), as well as life satisfaction in university students through the mediation of sEffic (X. Zhang & Ju, 2023) and academic performance in university students through the mediation of sEffic (Brouwer et al., 2016). Therefore, the following hypothesis is proposed:

**H5.**  *BSC has an indirect and negative effect on Dsym through the mediation of SES, IS, sEffic, sEstee, and ASsym*.

**Figure 1:**
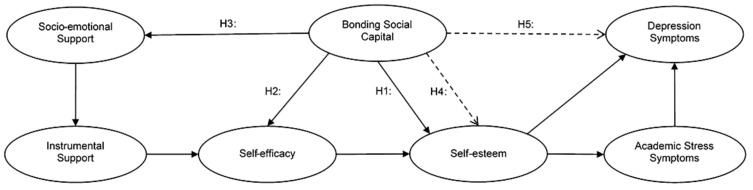
Structural model. Note: Segmented lines represent indirect relations.

To visualize the proposed hypotheses, Figure 1 presents the path model corresponding to the theoretical framework.

## 2. Materials and Methods

### 2.1. Research Design

The study uses a cross-sectional design to investigate the relationship between BSC and Dsym with the serial mediation of SES, IS, sEffic, sEstee, and ASsym in university students.

### 2.2. Participants and Procedure

The study sample was selected from a population of 49,332 students of the Major, Royal, and Pontifical Saint Francis Xavier of Chuquisaca University [Universidad Mayor Real y Pontificia de San Francisco Xavier de Chuquisaca] (USFX). Whereas the minimum sample required was 155 participants (significance level α = 0.05 y minimum path coefficients p_min_ 0.11–0.2) (Hair et al., 2022), a sample of 250 students was taken using stratified random sampling (considering gender and faculty) (Hernández-Sampieri & Mendoza Torres, 2018), 33 of which were excluded during data cleaning (due to entire surveys missing) (Hair et al., 2022), resulting in a final sample of 217 participants, whose ages ranged from 17 to 21 years, with an average of 19 years. Males numbered 102 (47.00%), and 115 (53.00%) were female.

The study was approved by the Ethics and Bioethics Committee of the Medicine Faculty of the Major University of San Andrés [Universidad Mayor de San Andres] (Ethical Endorsement: H.R. MED-2066/2023). Subsequently, authorization was obtained from the USFX, and its classrooms were used for data collection. Also, students were informed about the research characteristics and the voluntary nature of their participation; those who agreed to participate answered a battery of questionnaires and tests—it demanded an hour on average—between 10 October 2023 and 31 March 2024.

### 2.3. Instruments

Instrument selection was based on three essential methodological criteria. First, priority was given to aligning the scales with the theoretical definitions of the assessed variables, ensuring conceptual coherence between the constructs and their measurement. This principle is exemplified by the use of the Systemic Cognitive Inventory of Academic Stress SV-21 (SISCO SV-21) to evaluate academic stress (ASsym), grounded in the cognitive theory proposed by its author (Barraza-Macías, 2018). Second, preference was given to scales previously adapted and validated into Spanish with demonstrated reliability and validity in university populations. Examples include the Spanish-language version of the Medical Outcomes Study Social Support Scale (MOS-SSS) for social support (SES) and instrumental support (IS) (Revilla Ahumada et al., 2005) and the Rosenberg Self-Esteem Scale (Martín-Albo et al., 2007). Finally, their recent application in Bolivian research (Castro Torres et al., 2022, 2023) was considered, which supports their contextual relevance and comparability in the local setting. This triangulation of theoretical, psychometric, and contextual criteria ensures the robustness and validity of the measurements conducted.

BSC was evaluated using 8 items of the Personal Social Capital Scale 16 (PSCS-16), divided into four components: (1) reciprocity; (2) trust in network members; (3) network size; and (4) ownership of resources. Responses to each item were rated on a 5-point Likert scale. The original scale α is 0.90, with an adequate level of internal and criterion validity (P. Wang et al., 2014).

SES was evaluated with the emotional support dimension (8 items) and affective support (3 items) of the Spanish version of the Medical Outcomes Study Social Support Scale (MOS-SSS); IS was evaluated with the same-name dimension (4 items) of the MOS-SSS. All items were rated on a 5-option Likert scale. The original scale α is 0.941, with an adequate level of internal and criterion validity (Revilla Ahumada et al., 2005).

To asses sEffic, the Spanish version of the Perceived Stress Scale (PSS-14) was used, which consists of 7 items, rated on a 5-option Likert scale. The α of the original scale is 0.81, with an adequate level of criterion validity (Calderón Carvajal et al., 2017; Díaz-Corchuelo et al., 2015; Remor, 2006).

The Spanish version of the Rosenberg Self-Esteem Scale (10 items, rated on a Likert scale of 4 points) was employed to evaluate sEstee. The original scale α is 0.88, with an adequate level of criterion validity (Martín-Albo et al., 2007).

The ASsym level was evaluated through the same-name dimension of the Cognitive Systemic Inventory of Academic Stress SV-21 (SISCO SV-21), which consists of seven items rated on a 6-option Likert scale. The α of the original scale is 0.829, with an adequate level of convergent and discriminant validity (Barraza-Macías, 2018).

Finally, the Dsym level was evaluated through the Spanish version of the Beck Depression Inventory—Second Edition (BDI-II), which consists of 21 items, rated on a 4-option Likert scale. The original scale α varies between 0.87 and 0.89 in university students, with an adequate level of convergent and discriminatory validity (Beck et al., 2011).

### 2.4. Statistical Data Analysis

To analyze and evaluate the proposed model, partial least squares structural equations modeling (PLS-SEM) was used, given the characteristics of the data and the complexity of the model (Hair et al., 2022). Statistical analyses were performed using the SmartPLS program (Version 3.3.9) (Ringle et al., 2022) according to the parameters suggested by Hair et al. (2022). Also, since the BSC variable is a higher-order construct, the two-stage disjoint approach for higher-order constructs was used (Sarstedt et al., 2019).

The research model specified in the research model section consists of one exogenous latent variable, bonding social capital (BSC), and six endogenous latent variables, socio-emotional support (SES), instrumental support (IS), self-efficacy (sEffic), self-esteem (sEstee), academic stress symptoms (ASym), and depression symptoms (Dsym). Each construct operates under a reflective measurement model, with the following breakdown of indicators: 1. BSC: Four primary indicators, each measured via two reflective sub-indicators (reciprocity, trust in network members, network size, and ownership of resources); 2. SES: Eleven reflective indicators; 3. IS: Four reflective indicators; 4. sEffic: Seven reflective indicators; 5. sEstee: Ten reflective indicators; 6. ASym: Seven reflective indicators; and 7. Dsym: Twenty-one reflective indicators.

Analysis and evaluation followed two steps. First, the measurement model evaluation includes (a) indicator reliability by factorial loading (λ ≥ 0.40); (b) internal consistency of the reliability coefficient of the Dijkstra–Henseler Rho index (ρA ≥ 0.60 and <0.95); (c) convergent validity by the average variance extracted (AVE ≥ 0.5); and (d) discriminant validity by means of the proportion of heterotrait–monotrait correlations (HTMT ≤ 0.85 or HTMT ≤ 0.90, 95% IQ ≠ 1). Second is the structural model evaluation, considering (a) the collinearity by variance inflation factor (VIF < 5); (b) relevance and significance of path coefficients (β), including algebraic sign, magnitude (β ≥ 0.1), and the values of β through bias-corrected and accelerated bootstrap confidence intervals (BCa), with a confidence level of 5%, not including zero (95% ≠ 0 CI), obtained with a bootstrap of 10,000 samples; (c) in-sample predictive power through the coefficient of determination (R^2^ ≥ 0.1); and (d) out-of-sample predictive power, through the PLS predict procedure of the model’s key endogenous constructs’ indicators, including 1. the latent variables through the Stone–Geisser predict index (Q^2^_predict_ > 0) and 2. A comparison of the root mean square error (RMSE) values from the PLS-SEM analysis with the linear regression model (LM) values for each indicator of the key target constructs (RMSE-PLS < RMSE-LM) (Cepeda Carrión et al., 2017; Chin, 1998; Falk & Miller, 1992; Guenther et al., 2023; Hair et al., 2022; Ringle et al., 2023; Sarstedt et al., 2019).

## 3. Results

**Figure 2:**
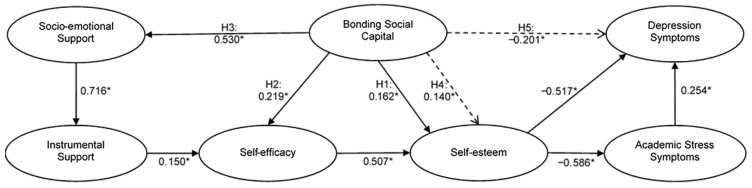
Empirical model: Results of the structural model. Note: Segmented lines represent indirect relations. * CI 95% ≠ 0.

The initial evaluation of the model determined the elimination of six of the seventy-two original indicators, since the metric reliability values of the latent variables to which they belonged showed values above 0.95. The excluded indicators were bdi2_10, bdi2_16, bdi2_18, and bdi2_21 from the depression symptoms (Dsym) construct and ss_ae_17 and ss_ae_19 from the socio-emotional support (SES) construct. With the remaining sixty-six indicators, the final analysis of the model was carried out, with the results presented below, as well as a graphic representation in Figure 2.

### 3.1. Measurement Model (Outer Model)

Evaluation of the measurement model was carried out through indicator reliability by factorial loadings (λ), internal consistency by means of the Dijkstra–Henseler Rho index reliability coefficient of the Dijkstra–Henseler Rho index (ρA), convergent validity by average variance extracted (AVE), and discriminant validity by means of the of the proportion of heterotrait–monotrait correlations (HTMT). As can be seen in Table 1, in relation to indicator reliability, fifty-six indicators have an optimal level (λ ≥ 0.708), and ten (bdi2_11, bdi2_17, bdi2_5, bdi2_6, bdi2_9, sEffic_13, sEffic_5, sEstee_5, sEstee_8, and sisco_s_15) have an acceptable level (λ ≥ 0.40). For internal consistency, all constructs have a satisfactory level (ρA ≥ 0.707). With respect to convergent validity, all constructs have an acceptable level (AVE ≥ 0.50). Similarly, all items have an optimal level of discriminant validity (HTMT ≤ 0.85, 95% CI ≠ 1), as seen in Appendix A.1.

### 3.2. Structural Model (Inner Model)

Evaluation of the structural model was carried out through (a) collinearity by VIF, (b) relevance and significance of path coefficients by β, (c) in-sample predictive power through the R^2^, and (d) out-of-sample predictive power through the Q^2^_predict_, as well as with the comparison of the RMSE values from the PLS-SEM analysis with the LM values for each indicator of the key target constructs. An evaluation of the results of the structural model demonstrates no collinearity issues (see Appendix A.2) because all values are acceptable (VIF < 5). Likewise, as seen in Table 2, the results demonstrate that BSC has a positive and direct influence on sEstee (H1) (β = 0.162, 95% CI [0.058, 0.264]); on the levels of perceived sEffic (H2) (β = 0.219, 95% CI [0.082, 0.332]); and on levels of SES (H3) (β = 0.530, 95% CI [0.427, 0.608]).

The analysis of mediation hypotheses 4 and 5 was carried out through the evaluation of indirect effects. The results of the total indirect effect and those of the specific indirect effects verify that the BSC has an indirect, relevant, and significant positive effect on sEstee (H4) (β = 0.140, 95% CI [0.074, 0.203]) by two routes. Likewise, BSC has an indirect, relevant, and significant negative effect on Dsym (H5) (β = −0.201, 95% CI [−0.266, −0.216]) through six routes.

The five hypothesized relationships among the constructs are relevant and statistically significant and coincide with the algebraic sign raised. Therefore, hypotheses 1, 2, 3, 4, and 5 were supported and accepted.

As shown in Table 3, the model has internal and external predictive relevance, since the value of the coefficients of determination and the Stone–Geisser predictive indices of the constructs exceed the cut-off value (R^2^ ≥ 0.1 and Q^2^_predict_ > 0). Also, the results from the comparison of the RMSE values from the PLS-SEM analysis with the LM values for each indicator of the key objective constructs show that all values of the constructs in the PLS-SEM analysis are lower than the LM values, so the model has high predictive power.

## 4. Discussion

The high prevalence of depressive symptoms (Dsym) among university students, associated with their impact on academic performance and quality of life, highlights the relevance of exploring protective mechanisms such as social capital. This study proposed and validated an integrative model explaining how bonding social capital (BSC) indirectly influences Dsym through the mediation of socio-emotional support (SES), instrumental support (IS), self-efficacy (sEffic), academic stress symptoms (ASsym), and self-esteem (sEstee).

The results support the proposed hypotheses. BSC has direct positive effects on self-esteem (sEstee), self-efficacy (sEffic), and socio-emotional support (SES), as well as an indirect negative effect on Dsym, mediated by SES, instrumental support (IS), sEffic, sEstee, and academic stress symptoms (ASsym). This chain of effects suggests that BSC acts as a structural resource that facilitates the activation of social support, a functional process, which, in turn, strengthens internal psychological resources (sEffic and sEstee). These resources buffer the impact of academic stress and reduce vulnerability to Dsym, aligning with related theories indicating that one mechanism linking social capital to mental health is its influence on psychosocial processes, i.e., as a source of sEffic and sEstee, which reduce Dsym (Kawachi & Berkman, 2014; Thoits, 1985, 2011).

These findings are supported by prior research demonstrating that BSC has a positive relationship with sEstee (Aziz et al., 2024; Clark, 2016), with sEffic (Brouwer et al., 2016; Castro Torres et al., 2022, 2023; Y. Liu & Ngai, 2020), and with social support (Castro Torres et al., 2022, 2023; Yoo, 2016, 2018). Similarly, other studies have shown that sEffic positively influences sEstee (Bayani, 2016; Caprara et al., 2013; Fu et al., 2019).

As the results show, the model exhibits internal and external predictive relevance, as evidenced by the coefficients of determination (R^2^ ≥ 0.1) and the Stone–Geisser indices (Q^2^_predict_ > 0) exceeding the established thresholds. Additionally, the comparison between the PLS-SEM RMSE values and the LM (linear model) values for key constructs’ indicators reveals that all PLS-SEM values are lower than the LM values; this confirms that the model not only meets statistical validity criteria but also demonstrates high predictive power, outperforming other methodological alternatives in explaining the studied mechanisms.

Furthermore, the findings align with previous studies identifying that social capital reduces Dsym in university students (Backhaus et al., 2020, 2022; Sotaquirá et al., 2022; Zhao et al., 2024), as well as research showing that academic stress positively influences Dsym in this population (Backhaus et al., 2020; B. Chen et al., 2024; Deng et al., 2022). Finally, prior studies have demonstrated that sEstee reduces stress and Dsym in university students (Bayani, 2016; Huang et al., 2023; X. Liu et al., 2024; Saleh et al., 2017).

The findings not only align with prior research but also expand the literature by demonstrating a sequential mediation mechanism integrating psychosocial variables. Unlike studies analyzing isolated effects, this model highlights the interdependence between social capital, functional (social) support, and psychological resources. However, it contrasts with work reporting the negative effects of certain social capital components (Peng et al., 2019), which may stem from cultural differences in perceptions of social networks or the influence of other mediators.

Theoretically, the study validates the need to distinguish between social capital (as a structural resource) and social support (as a functional process), as proposed by Lin and Ensel (1989) and Castro Torres et al. (2022, 2023). It also integrates multidisciplinary frameworks (e.g., social psychology and public health) to explain depression in academic contexts. Notably, the study identifies sEstee as the predominant final mediator over sEffic. This hierarchy can be explained by two complementary perspectives. First, the nature of the constructs offers an interpretive key. sEffic, i.e., beliefs about one’s ability to organize and execute goal-oriented actions (Bandura, 1993; Maddux & Kleiman, 2016), operates primarily in the domain of specific competencies. In contrast, sEstee, i.e., a global self-attitude integrating cognitive (self-evaluation) and affective (emotional appraisal) components (Rosenberg et al., 1995; Maddux & Kleiman, 2016), acts as an emotional filter for perceived academic stress and its eventual translation into depressive symptoms. This conceptual distinction aligns with prior findings identifying sEffic as an antecedent of sEstee (Bayani, 2016; Caprara et al., 2013; Fu et al., 2019), suggesting a hierarchical relationship where sEstee synthesizes diverse efficacy experiences, consistent with Thoits’ social support theory (Thoits, 1985, 2011). A second explanatory factor emerges when contrasting our results with prior studies, e.g., Castro Torres et al. (2022), which analyzed academic stress symptoms as the final outcome and dismissed sEstee as a mediator due to a lack of statistical support. This divergence suggests that the mediating role of constructs may vary depending on the outcome measured. While academic stress may link more directly to perceptions of capability (sEffic), depression—the final variable in our model—may align more with global affective–evaluative mechanisms (sEstee).

The study’s main limitations include (a) the cross-sectional design, which precludes causal inferences and complicates establishing the directionality of proposed relationships, particularly the bidirectional link between sEstee and Dsym, implying that depression may reduce sEstee. (b) Data collection from a single university limits generalizability. (c) The effect sizes, while statistically significant, remain modest, indicating the potential impact of unmeasured variables (e.g., personality traits). Furthermore, the identified mediation pathways may be prone to omitted variables bias: e.g., factors such as neuroticism, prior traumatic experiences, or coping family dynamics could simultaneously influence perceptions of social support, self-esteem, and depressive symptoms, potentially confounding the observed relationships. To disentangle these effects and clarify social capital’s specific role in mitigating depression, future longitudinal and/or experimental studies should integrate such variables into their designs, replicating the model across diverse contexts and using longitudinal approaches that would strengthen causal inferences and validate the proposed framework. These steps would refine the mediation pathways and help to isolate the mechanisms through which social capital buffers depressive symptoms.

## 5. Conclusions

This study demonstrates that bonding social capital (BSC) is indirectly associated with reduced depressive symptoms in university students through a sequential mechanism. This process integrates socio-emotional and instrumental support, self-efficacy, self-esteem, and reduced academic stress, highlighting self-esteem’s central role as a key mediator.

The contributions of this research encompass two main dimensions. Theoretically, it validates an integrative model, clarifying the relationship between social capital and depression, overcoming fragmented approaches by articulating key variables into a coherent structure. This framework not only confirms the conceptual distinction between social capital and social support but also identifies self-esteem as a critical factor in depressive symptomatology, integrating prior findings on self-efficacy in line with Thoits’ (1985, 2011) theory. Practically, the model’s predictive robustness is supported by strong statistical indicators (R^2^, Q^2^_predict_, and RMSE comparisons), suggesting its capacity to generalize patterns beyond the analyzed sample. These metric properties, alongside the theoretical consistency of identified relationships, provide a solid foundation for designing evidence-based interventions.

However, these findings must be contextualized within methodological limitations. While the proposed model represents a pioneering advance in addressing the phenomenon systemically, its replication in culturally diverse samples with longitudinal designs would allow for causal relationships to be established and its applicability assessed across institutional contexts (external validation). Future research should further verify and expand the proposed framework and explore additional mediators or moderators.

Despite these limitations, the study offers an innovative theoretical–methodological framework for analyzing the interaction between social determinants and mental health. Its results have practical implications. Interventions aimed at strengthening social capital, combined with strategies to enhance self-esteem (once the model is validated), could optimize depression prevention in university settings. This would not only contribute to student well-being but also their academic success, underscoring the importance of multilevel approaches to mental health.

## Figures and Tables

**Table 1:** Evaluation of the measurement model.

Construct	Indicator	λ ^a^	ρA ^b^	AVE ^c^
First-order constructs				
BSC-Tr			0.809 ***	0.832 ***
	Item 3	0.924 ***		
	Item 4	0.900 ***		
BSC-RP			0.752 ***	0.734 ***
	Item 5	0.786 ***		
	Item 6	0.922 ***		
BSC-Re			0.810 ***	0.840 ***
	Item 7	0.912 ***		
	Item 8	0.920 ***		
BSC-NS			0.736 ***	0.777 ***
	Item 1	0.906 ***		
	Item 2	0.856 ***		
Socio-emotional support			0.945 ***	0.693 ***
	ss_aa_10	0.832 ***		
	ss_aa_20	0.794 ***		
	ss_aa_6	0.824 ***		
	ss_ae_13	0.837 ***		
	ss_ae_16	0.856 ***		
	ss_ae_3	0.831 ***		
	ss_ae_4	0.826 ***		
	ss_ae_8	0.827 ***		
	ss_ae_9	0.867 ***		
Instrumental support			0.897 ***	0.759 ***
	ss_ai_12	0.877 ***		
	ss_ai_15	0.863 ***		
	ss_ai_2	0.838 ***		
	ss_ai_5	0.906 ***		
Self-esteem			0.923 ***	0.563 ***
	sEstee_1	0.762 ***		
	sEstee_10	0.812 ***		
	sEstee_2	0.803 ***		
	sEstee_3	0.771 ***		
	sEstee_4	0.732 ***		
	sEstee_5	0.640 ***		
	sEstee_6	0.807 ***		
	sEstee_7	0.731 ***		
	sEstee_8	0.518 ***		
	sEstee_9	0.865 ***		
Self-efficacy			0.871 ***	0.548 ***
	sEffic_10	0.767 ***		
	sEffic_13	0.633 ***		
	sEffic_4	0.735 ***		
	sEffic_5	0.702 ***		
	sEffic_6	0.788 ***		
	sEffic_7	0.773 ***		
	sEffic_9	0.773 ***		
Academic stress symptoms			0.910 ***	0.626 ***
	sisco_s_10	0.767 ***		
	sisco_s_11	0.870 ***		
	sisco_s_12	0.858 ***		
	sisco_s_13	0.796 ***		
	sisco_s_14	0.772 ***		
	sisco_s_15	0.669 ***		
	sisco_s_16	0.787 ***		
Depression symptoms			0.942 ***	0.509 ***
	bdi2_1	0.721 ***		
	bdi2_11	0.615 ***		
	bdi2_12	0.719 ***		
	bdi2_13	0.794 ***		
	bdi2_14	0.769 ***		
	bdi2_15	0.728 ***		
	bdi2_17	0.677 ***		
	bdi2_19	0.704 ***		
	bdi2_2	0.708 ***		
	bdi2_20	0.741 ***		
	bdi2_3	0.721 ***		
	bdi2_4	0.702 ***		
	bdi2_5	0.697 ***		
	bdi2_6	0.673 ***		
	bdi2_7	0.718 ***		
	bdi2_8	0.762 ***		
	bdi2_9	0.664 ***		
Second-order constructs				
Bonding social capital			0.891 ***	0.749 ***
	BSC-Tr	0.754 ***		
	BSC-RP	0.577 ***		
	BSC-Re	0.841 ***		
	BSC-NS	0.746 ***		

^a^ Factorial loadings, ^b^ Dijkstra–Henseler Rho, ^c^ Average variance extracted. *** *p* ≤ 0.001, based on one-tailed *t* test (10,000). Note. BSC-Tr = bonding social capital—dimension trust; BSC-RP = bonding social capital—dimension resources property; BSC-Re = bonding social capital—dimension reciprocity; BSC-NS = bonding social capital—dimension net size.

**Table 2:** Structural model assessment. Direct and indirect effects (hypothesized relationships).

Hypothesis	Relation	(β)	CI 95% (^a^)		Conclusions
			LL	UL	
Direct effects					
H1 (+)	BSC → sEstee	0.162	0.058	0.264	Supported
H2 (+)	BSC → sEffic	0.219	0.082	0.332	Supported
H3 (+)	BSC → SES	0.530	0.427	0.608	Supported
Total indirect effect 1					
H4 (+)	BSC → sEstee	0.140	0.074	0.203	Supported
Specific indirect effects 1					
	BSC → sEffic → sEstee	0.111	0.041	0.179	Supported
	BSC → SES → IS → sEffic → sEstee	0.029	0.006	0.058	Supported
Total indirect effect 2					
H5 (−)	BSC → Dsym	−0.201	−0.266	−0.126	Supported
Specific indirect effects 2					
	BSC → sEstee → Dsym	−0.084	−0.142	−0.031	Supported
	BSC → sEstee → ASsym→ Dsym	−0.024	−0.047	−0.009	Supported
	BSC→ sEffic → sEstee → Dsym	−0.057	−0.095	−0.021	Supported
	BSC→ sEffic → sEstee → ASsym → Dsym	−0.016	−0.032	−0.005	Supported
	BSC→ SES → IS → sEffic → sEstee → Dsym	−0.015	−0.032	−0.003	Supported
	BSC→ SES → IS → sEffic → sEstee → ASsym → Dsym	−0.004	−0.010	−0.001	Supported

Note. CI = confidence interval; LL = lower limit; UL = upper limit; BSC = bonding social capital; SES = socio-emotional support; IS = instrumental support; sEffic = self-efficacy; sEstee = self-esteem; ASsym = academic stress symptoms; Dsym = depression symptoms. ^a^ Bias-corrected and accelerated (BCa) bootstrap confidence intervals for 5% probability of error (α = 0.05).

**Table 3:** Structural model assessment. Evaluation of in-sample predictive power (R^2^) and out-of-sample predictive power (Q^2^_predict_ and RMSE-PLS, RMSE-LM).

Construct	Indicator Key	R^2^	Q^2^_predict_	RMSE (^a^)		Conclusions
				PLS	LM	
IS		0.512	0.196			Supported
SES		0.281	0.270			Supported
sEffic		0.100	0.069			Supported
Dsym		0.486	0.036			Supported
	bdi2_11			0.834	0.842	Supported
	bdi2_12			0.802	0.808	Supported
	bdi2_8			0.930	0.940	Supported
	bdi2_1			0.668	0.678	Supported
	bdi2_15			0.784	0.796	Supported
	bdi2_7			0.880	0.888	Supported
	bdi2_2			0.755	0.757	Supported
	bdi2_6			0.857	0.868	Supported
	bdi2_14			0.890	0.901	Supported
	bdi2_19			0.788	0.790	Supported
	bdi2_20			0.873	0.880	Supported
	bdi2_3			0.802	0.808	Supported
	bdi2_13			0.933	0.944	Supported
	bdi2_5			0.745	0.754	Supported
	bdi2_9			0.681	0.692	Supported
	bdi2_4			0.729	0.733	Supported
	bdi2_17			0.750	0.760	Supported
ASsym		0.343	0.027			Supported
	sisco_s_10			1.354	1.364	Supported
	sisco_s_13			1.172	1.189	Supported
	sisco_s_16			1.235	1.243	Supported
	sisco_s_15			1.288	1.289	Supported
	sisco_s_12			1.330	1.349	Supported
	sisco_s_14			1.356	1.357	Supported
	sisco_s_11			1.350	1.363	Supported
sEstee		0.330	0.084			Supported
	sEstee_8			0.765	0.775	Supported
	sEstee_3			0.650	0.660	Supported
	sEstee_7			0.695	0.707	Supported
	sEstee_4			0.686	0.697	Supported
	sEstee_5			0.834	0.846	Supported
	sEstee_6			0.870	0.873	Supported
	sEstee_9			0.855	0.862	Supported
	sEstee_1			0.738	0.741	Supported
	sEstee_10			0.753	0.761	Supported
	sEstee_2			0.823	0.832	Supported

Note. R^2^ = coefficient of determination; Q^2^_predict_ = Stone–Geisser index predict; RMSE = root mean square error; PLS = PLS-SEM prediction errors; LM = linear regression model prediction errors; SES = socio-emotional support; IS = instrumental support; sEffic = self-efficacy; ASsym = academic stress symptoms; Dsym = depression symptoms. ^a^ PLSpredict procedure of model’s key endogenous constructs’ indicators, *k*-fold cross-validation = 10, Number of Repetitions = 10.

## Data Availability

The data presented in this study are available upon request from the corresponding author. The data are not publicly available due to privacy and confidentiality.

## Abbreviations

The following abbreviations are used in this manuscript:

ASsym	Academic stress symptoms
AVE	Average variance extracted
BSC	Bonding social capital
BSC-Tr	Bonding social capital—dimension trust
BSC-RP	Bonding social capital—dimension resources property
BSC-Re	Bonding social capital—dimension reciprocity
BSC-NS	Bonding social capital—dimension net size.
Dsym	Depression symptoms
HTMT	Heterotrait–monotrait
IS	Instrumental support
LM	Linear regression model
PLS-SEM	Partial least squares structural equations modeling
RMSE	Root mean square error
sEffic	Self-efficacy
SES	Socio-emotional support
sEstee	Self-esteem
USFX	Major, Royal, and Pontifical Saint Francis Xavier of Chuquisaca University [Universidad Mayor Real y Pontificia de San Francisco Xavier de Chuquisaca]
VIF	Variance inflation factor

## Appendix A

### Appendix A.1

**Table A1:** Evaluation of measurement model: discriminant validity (HTMT).

Construct	1	2	3	4	5	6	7	8	9	10	11
First-order constructs											
(1) IS	—										
(2) BSC-NS	0.387 *										
(3) BSC-Tr	0.400 *	0.472 *									
(4) BSC-RP	0.327 *	0.470 *	0.448 *								
(5) BSC-Re	0.474 *	0.568 *	0.669 *	0.510 *							
(6) Depression	0.214 *	0.264 *	0.203 *	0.077 *	0.166 *						
(7) SES	0.776 *	0.472 *	0.436 *	0.278 *	0.573 *	0.246 *					
(8) sAS	0.101 *	0.229 *	0.191 *	0.077 *	0.148 *	0.594 *	0.120 *				
(9) Self-efficacy	0.280 *	0.322 *	0.190 *	0.187 *	0.292 *	0.463 *	0.356 *	0.310 *			
(10) Self-esteem	0.172 *	0.339 *	0.248 *	0.142 *	0.297 *	0.705 *	0.306 *	0.631 *	0.609 *	—	
Second-order constructs											
(11) BSC	0.553 *					0.241 *	0.615 *	0.222 *	0.346 *	0.359 *	—

* CI 95% ≠ 1.

### Appendix A.2

**Table A2:** Evaluation of structural model: collinearity (VIF values).

Construct	1	2	3	4	5	6	7
(1) IS						1.256	
(2) BSC				1.000		1.256	1.089
(3) Dsym							
(4) SES	1.000						
(5) ASsym			1.522				
(6) Self-efficacy							1.089
(7) Self-esteem			1.522		1.000		

## References

[B1-ejihpe-15-00083] Adler P. S., Kwon S.-W. (2002). Social Capital: Prospects for a New Concept. Academy of Management Review.

[B2-ejihpe-15-00083] Akhtar P., Ma L., Waqas A., Naveed S., Li Y., Rahman A., Wang Y. (2020). Prevalence of depression among university students in low and middle income countries (LMICs): A systematic review and meta-analysis. Journal of Affective Disorders.

[B3-ejihpe-15-00083] American Psychiatric Association (2013). Diagnostic and statistical manual of mental disorders (DSM-5 (R)).

[B4-ejihpe-15-00083] Arévalo García E., Castillo-Jimenez D. A., Cepeda I., López Pacheco J., Pacheco López R. (2019). Ansiedad y depresión en estudiantes universitarios: Relación con rendimiento académico. Interdisciplinary Journal of Epidemiology and Public Health.

[B5-ejihpe-15-00083] Auerbach R. P., Mortier P., Bruffaerts R., Alonso J., Benjet C., Cuijpers P., Demyttenaere K., Ebert D. D., Green J. G., Hasking P., Murray E., Nock M. K., Pinder-Amaker S., Sampson N. A., Stein D. J., Vilagut G., Zaslavsky A. M., Kessler R. C., WHO WMH-ICS Collaborators (2018). WHO world mental health surveys international college student project: Prevalence and distribution of mental disorders. Journal of Abnormal Psychology.

[B6-ejihpe-15-00083] Aziz M., Gupta S., Mir S. M., Bashir I., Khurshid S., Amin F. (2024). Influence of family social capital on the psychological well-being of working women: Mediating role of self-esteem and moderating role of sense of coherence. Family Journal.

[B7-ejihpe-15-00083] Backhaus I., Borges C., Baer A. de P., Monteiro L. Z., Torre G. L., Varela A. R. (2022). Association between social capital indicators and depressive symptoms among Brazilian university students. Ciencia & Saude Coletiva.

[B8-ejihpe-15-00083] Backhaus I., Varela A. R., Khoo S., Siefken K., Crozier A., Begotaraj E., Fischer F., Wiehn J., Lanning B. A., Lin P.-H., Jang S.-N., Monteiro L. Z., Al-Shamli A., La Torre G., Kawachi I. (2020). Associations between social capital and depressive symptoms among college students in 12 countries: Results of a cross-national study. Frontiers in Psychology.

[B9-ejihpe-15-00083] Bandura A. (1993). Perceived self-efficacy in cognitive development and functioning. Educational Psychologist.

[B10-ejihpe-15-00083] Bandura A. (2010). Exercise of personal and collective efficacy in changing societies. Self-efficacy in changing societies.

[B11-ejihpe-15-00083] Barraza-Macías A., Ramos Escamilla M. (2018). INVENTARIO SISCO SV-21. Inventario sistémico cognoscitivista para el estudio del estrés académico. Segunda versión de 21 ítems.

[B12-ejihpe-15-00083] Bayani A. A. (2016). The effect of self-esteem, self-efficacy and family social support on test anxiety in elementary students: A path model. International Journal of School Health.

[B13-ejihpe-15-00083] Beck A. T., Sanz Fernández J., Brown G. K., Steer R. A., Vázquez Valverde C. (2011). BDI-II: Inventario de depresión de Beck-II: Manual/Aaron T. Beck, Robert A. Steer, Gregory K. Brown; adaptación española Jesús Sanz, Carmelo Vázquez.

[B14-ejihpe-15-00083] Bourdieu P. (2001). Poder, derecho y clases sociales.

[B15-ejihpe-15-00083] Brouwer J., Jansen E., Flache A., Hofman A. (2016). The impact of social capital on self-efficacy and study success among first-year university students. Learning and Individual Differences.

[B16-ejihpe-15-00083] Buelga S., Musitu G., Musitu G., Buelga S., Vera A., Ávila M. E., Arango C. (2009). Orientaciones clínico-comunitarias. Psicología social comunitaria.

[B17-ejihpe-15-00083] Calderón Carvajal C., Gomez N., López F., Otárola N., Briceño M. (2017). Estructura factorial de la escala de estrés percibido (PSS) en una muestra de trabajadores chilenos. Salud y Sociedad.

[B18-ejihpe-15-00083] Caprara G. V., Alessandri G., Barbaranelli C., Vecchione M. (2013). The longitudinal relations between self-esteem and affective self-regulatory efficacy. Journal of Research in Personality.

[B19-ejihpe-15-00083] Castro Torres M. E., Vargas-Piérola P. M., Pinto C. F., Alvarado R. (2022). Serial mediation model of social capital effects over academic stress in university students. European Journal of Investigation in Health Psychology and Education.

[B20-ejihpe-15-00083] Castro Torres M. E., Vargas-Piérola P. M., Pinto C. F., Alvarado R. (2023). Multiple sequential mediation model of the effect of social capital investment on academic stress. International Journal of Educational Research Open.

[B21-ejihpe-15-00083] Cepeda Carrión G., Nitzl C., Roldán J. L. (2017). Mediation analyses in partial least squares structural equation modeling: Guidelines and empirical examples. Partial least squares path modeling.

[B22-ejihpe-15-00083] Chen B., Wang W., Yang S. (2024). The relationship between academic stress and depression among college students during the COVID-19 pandemic: A cross-sectional study from China. BMC Psychiatry.

[B23-ejihpe-15-00083] Chen X., Stanton B., Gong J., Fang X., Li X. (2009). Personal social capital scale: An instrument for health and behavioral research. Health Education Research.

[B24-ejihpe-15-00083] Chin W. W. (1998). Commentary: Issues and Opinion on Structural Equation Modeling. MIS Quarterly.

[B25-ejihpe-15-00083] Clark C. V. (2016). The anonymous network: Perceptions of social capital and well-being among college students on yik yak. Master’s thesis.

[B26-ejihpe-15-00083] Cohen S., Gottlieb B. H., Underwood L. G. (2000). Social Relationships and Health. Social support measurement and intervention.

[B27-ejihpe-15-00083] Córdova Olivera P., Gasser Gordillo P., Naranjo Mejía H., La Fuente Taborga I., Grajeda Chacón A., Sanjinés Unzueta A. (2023). Academic stress as a predictor of mental health in university students. Cogent Education.

[B28-ejihpe-15-00083] Dela Cruz C. J. B. C., Girlie E. A. (2022). Social support, self-efficacy, and spirituality of adolescents: A structural equation model of their personal resilience during a pandemic. American Journal of Multidisciplinary Research and Innovation.

[B29-ejihpe-15-00083] Deng Y., Cherian J., Khan N. U. N., Kumari K., Sial M. S., Comite U., Gavurova B., Popp J. (2022). Family and academic stress and their impact on students’ depression level and academic performance. Frontiers in Psychiatry.

[B30-ejihpe-15-00083] Díaz-Corchuelo A., Cordón-Pozo E., Rubio-Herrera R. (2015). Percepción de estrés en personal universitario. Diversitas: Perspectivas En Psicología.

[B31-ejihpe-15-00083] Falk R. F., Miller N. B. (1992). A Primer for soft modeling.

[B32-ejihpe-15-00083] Fernandes M. d. S. V., Mendonça C. R., da Silva T. M. V., Noll P. R. e. S., de Abreu L. C., Noll M. (2023). Relationship between depression and quality of life among students: A systematic review and meta-analysis. Scientific Reports.

[B33-ejihpe-15-00083] Fu W., Tang W., Xue E., Li J., Shan C. (2019). The mediation effect of self-esteem on job-burnout and self-efficacy of special education teachers in Western China. International Journal of Developmental Disabilities.

[B34-ejihpe-15-00083] Fuertes Eugenio A. M., Agost Felip M. R., Fuertes Fuertes I., Soto Personat G. (2013). Las aportaciones del apoyo social al capital social: Propuesta de un modelo integrado y convergente. CIRIEC-España. Revista de Economía Pública, Social y Cooperativa.

[B35-ejihpe-15-00083] Gannon B., Roberts J. (2020). Social capital: Exploring the theory and empirical divide. Empirical Economics.

[B36-ejihpe-15-00083] Guenther P., Guenther M., Ringle C. M., Zaefarian G., Cartwright S. (2023). Improving PLS-SEM use for business marketing research. Industrial Marketing Management.

[B37-ejihpe-15-00083] Hair J., Hult G. T. M., Ringle C. M., Sarstedt M. (2022). A primer on partial least squares structural equation modeling (PLS-SEM).

[B38-ejihpe-15-00083] Hernández-Sampieri R., Mendoza Torres C. P. (2018). Metodología de la investigación. Las rutas cuantitativa, cualitativa y mixta.

[B39-ejihpe-15-00083] Herrero Olaizola J., Musitu Ochoa G., Herrero Olaizola J., Cantera Espinosa L. M., Montenegro Martínez M. (2004). Capítuo VII. Redes sociales y apoyo social. Introducción a la psicología comunitaria.

[B40-ejihpe-15-00083] Holmgreen L., Tirone V., Gerhart J., Hobfoll S. E. (2017). Conservation of resources theory: Resource caravans and passageways in health contexts. The handbook of stress and health.

[B41-ejihpe-15-00083] Huang Y., Wongpakaran T., Wongpakaran N., Bhatarasakoon P., Pichayapan P., Worland S. (2023). Depression and its associated factors among undergraduate engineering students: A cross-sectional survey in Thailand. Healthcare.

[B42-ejihpe-15-00083] Kawachi I., Berkman L. F. (2014). Social capital, social cohesion, and health. Social epidemiology.

[B43-ejihpe-15-00083] Kawachi I., Subramanian S. V., Kim D. (2008). Social capital and health. Social capital and health.

[B44-ejihpe-15-00083] Lazarus R. S. (2001). Relational meaning and discrete emotions. Appraisal processes in emotion.

[B45-ejihpe-15-00083] Lazarus R. S., Folkman S. (1984). Stress, appraisal, and coping.

[B46-ejihpe-15-00083] Li W., Zhao Z., Chen D., Peng Y., Lu Z. (2022). Prevalence and associated factors of depression and anxiety symptoms among college students: A systematic review and meta-analysis. Journal of Child Psychology and Psychiatry, and Allied Disciplines.

[B47-ejihpe-15-00083] Li Z.-H., Wang J., Cheng X., Mao Y.-C., Zhang K.-D., Yu W.-J., Li Y.-Q., Huang K., Ding K., Yang X.-J., Hu C.-Y., Zhang X.-J. (2023). The role of self-esteem in the relationship between psychological capital and anxiety of left-behind experience college students during COVID-19 pandemic: An online study. Psychology Research and Behavior Management.

[B48-ejihpe-15-00083] Lin N. (2001). Social capital: Capital captured through Social Relations. Social capital: A theory of social structure and action.

[B49-ejihpe-15-00083] Lin N., Ensel W. M. (1989). Life stress and health: Stressors and resources. American Sociological Review.

[B50-ejihpe-15-00083] Liu X., Yuan Y., Gao W., Luo Y. (2024). Longitudinal trajectories of self-esteem, related predictors, and impact on depression among students over a four-year period at college in China. Humanities & Social Sciences Communications.

[B51-ejihpe-15-00083] Liu X.-Q., Guo Y.-X., Zhang W.-J., Gao W.-J. (2022). Influencing factors, prediction and prevention of depression in college students: A literature review. World Journal of Psychiatry.

[B52-ejihpe-15-00083] Liu Y., Ngai S. S.-Y. (2020). Social capital, self-efficacy, and healthy identity development among Chinese adolescents with and without economic disadvantages. Journal of Child and Family Studies.

[B53-ejihpe-15-00083] Maddux J. E., Kleiman E. M., Snyder C. R., Lopez S. J., Edwards L. M., Marques S. C. (2016). Self-efficacy.

[B54-ejihpe-15-00083] Martín-Albo J., Núñiez J. L., Navarro J. G., Grijalvo F. (2007). The rosenberg self-esteem scale: Translation and validation in university students. The Spanish Journal of Psychology.

[B55-ejihpe-15-00083] McEwen B. S., McEwen C. A. (2015). Social, psychological, and physiological reactions to stress. Emerging trends in the social and behavioral sciences.

[B56-ejihpe-15-00083] Mishi S., Sibanda K., Anakpo G. (2023). The concept and application of social capital in health, education and employment: A scoping review. Social Sciences.

[B57-ejihpe-15-00083] Mofatteh M. (2021). Risk factors associated with stress, anxiety, and depression among university undergraduate students. AIMS Public Health.

[B58-ejihpe-15-00083] Paula W. d., Breguez G. S., Machado E. L., Meireles A. L. (2020). Prevalence of anxiety, depression, and suicidal ideation symptoms among university students: A systematic review. Brazilian Journal of Health Review.

[B59-ejihpe-15-00083] Peng S., Yang X. Y., Rockett I. R. H. (2019). A typology of social capital and its mixed blessing for suicidal ideation: A multilevel study of college students. Social Science & Medicine.

[B60-ejihpe-15-00083] Putnam R. D. (2001). Bowling alone.

[B61-ejihpe-15-00083] Remor E. (2006). Psychometric properties of a European Spanish version of the Perceived Stress Scale (PSS). The Spanish Journal of Psychology.

[B62-ejihpe-15-00083] Revilla Ahumada L. d. l., Luna del Castillo J., Bailón Muñoz E., Medina Moruno I. (2005). Validación del cuestionario MOS de apoyo social en Atención Primaria. Medicina de Familia.

[B63-ejihpe-15-00083] Ringle C. M., Sarstedt M., Sinkovics N., Sinkovics R. R. (2023). A perspective on using partial least squares structural equation modelling in data articles. Data in Brief.

[B64-ejihpe-15-00083] Ringle C. M., Wende S., Becker J. M. (2022). SmartPLS *(Version 3.3.9)*.

[B65-ejihpe-15-00083] Roldán-Espínola L., Riera-Serra P., Roca M., García-Toro M., Coronado-Simsic V., Castro A., Navarra-Ventura G., Vilagut G., Alayo I., Ballester L., Blasco M. J., Almenara J., Cebrià A. I., Echeburúa E., Gabilondo A., Lagares C., Piqueras J. A., Soto-Sanz V., Mortier P., Kessler R. C. (2024). Depression and lifestyle among university students: A one-year follow-up study. The European Journal of Psychiatry.

[B66-ejihpe-15-00083] Rosenberg M., Schooler C., Schoenbach C., Rosenberg F. (1995). Global self-esteem and specific self-esteem: Different concepts, different outcomes. American Sociological Review.

[B67-ejihpe-15-00083] Saleh D., Camart N., Romo L. (2017). Predictors of stress in college students. Frontiers in Psychology.

[B68-ejihpe-15-00083] Sarstedt M., Hair J. F., Cheah J.-H., Becker J.-M., Ringle C. M. (2019). How to specify, estimate, and validate higher-order constructs in PLS-SEM. Australasian Marketing Journal (AMJ).

[B69-ejihpe-15-00083] Sheldon E., Simmonds-Buckley M., Bone C., Mascarenhas T., Chan N., Wincott M., Gleeson H., Sow K., Hind D., Barkham M. (2021). Prevalence and risk factors for mental health problems in university undergraduate students: A systematic review with meta-analysis. Journal of Affective Disorders.

[B70-ejihpe-15-00083] Sotaquirá L., Backhaus I., Sotaquirá P., Pinilla-Roncancio M., González-Uribe C., Bernal R., Galeano J. J., Mejia N., La Torre G., Trujillo-Maza E. M., Suárez D. E., Duperly J., Ramirez Varela A. (2022). Social capital and lifestyle impacts on mental health in university students in Colombia: An observational study. Frontiers in Public Health.

[B71-ejihpe-15-00083] Suárez-Montes N., Díaz-Subieta L. B. (2015). Estrés académico, deserción y estrategias de retención de estudiantes en la educación superior. Revista de Salud Publica.

[B72-ejihpe-15-00083] Tafet G. E. (2022). Neuroscience of stress: From neurobiology to cognitive, emotional and behavioral sciences.

[B73-ejihpe-15-00083] Thoits P. A. (1985). Social support and psychological well-being: Theoretical possibilities. Social support: Theory, research and applications.

[B74-ejihpe-15-00083] Thoits P. A. (2011). Mechanisms linking social ties and support to physical and mental health. Journal of Health and Social Behavior.

[B75-ejihpe-15-00083] VandenBos G. R. (2015). APA dictionary of psychology.

[B76-ejihpe-15-00083] Wade-Bohleber L. M., Duss C., Crameri A., von Wyl A. (2020). Associations of social and psychological resources with different facets of chronic stress: A study with employed and unemployed adolescents. International Journal of Environmental Research and Public Health.

[B77-ejihpe-15-00083] Wang P., Chen X., Gong J., Jacques-Tiura A. J. (2014). Reliability and validity of the personal social capital scale 16 and personal social capital scale 8: Two short instruments for survey studies. Social Indicators Research.

[B78-ejihpe-15-00083] Wang Y., Zhang S., Liu X., Shi H., Deng X. (2023). Differences in central symptoms of anxiety and depression between college students with different academic performance: A network analysis. Frontiers in Psychology.

[B79-ejihpe-15-00083] Watkins K., Hill E. M. (2018). The role of stress in the social support–mental health relationship. Journal of College Counseling.

[B80-ejihpe-15-00083] Yang L., Yuan J., Sun H., Zhao Y., Yu J., Li Y. (2023). Influencing factors of depressive symptoms among undergraduates: A systematic review and meta-analysis. PLoS ONE.

[B81-ejihpe-15-00083] Yoo C. (2016). Longitudinal relationship between academic stress and bonding social capital: Risk and protective roles of ‘bonding social capital and academic stress’ according to specific situations in south Korean adolescents. Child Indicators Research.

[B82-ejihpe-15-00083] Yoo C. (2018). Academic stress and mental health among adolescents: Applying a multi-dimensional stress coping model. Doctoral dissertation.

[B83-ejihpe-15-00083] Zhang J., Peng C., Chen C. (2024). Mental health and academic performance of college students: Knowledge in the field of mental health, self-control, and learning in college. Acta Psychologica.

[B84-ejihpe-15-00083] Zhang X., Ju S.-Y. (2023). What matters for life satisfaction of university students: The role of social capital and self-efficacy?. South Asian Journal of Social Sciences and Humanities.

[B85-ejihpe-15-00083] Zhao J., Nie L., Pan L., Pang M., Wang J., Zhou Y., Chen R., Liu H., Xu X., Zhou C., Li S., Kong F. (2024). Association between social capital, mental health, and digital health literacy among the university students in China: A multigroup analysis based on major difference. BMC Public Health.

